# Intracellular and Extracellular Metabolic Response of the Lactic Acid Bacterium *Weissella confusa* Under Salt Stress

**DOI:** 10.3390/metabo14120695

**Published:** 2024-12-10

**Authors:** Ali Wang, Qinqin Du, Xiaomin Li, Yimin Cui, Jiahua Luo, Cairong Li, Chong Peng, Xianfeng Zhong, Guidong Huang

**Affiliations:** 1 School of Food Science and Engineering, Foshan University, Foshan 528231, China; wangali526@163.com (A.W.); 2112208091@fosu.edu.cn (Q.D.); lixiaomin23@163.com (X.L.); cui_0129_211@163.com (Y.C.); luojiahua13@163.com (J.L.); crcr199301@163.com (C.L.); pc16293547@163.com (C.P.); 2 Guangdong Engineering Research Center for Traditional Fermented Food, Guangdong Engineering Research Center for Safety Control of Food Circulation, Foshan Engineering Research Center for Brewing Technology, Foshan Engineering Research Center for Agricultural Biomanufacturing, Foshan 528231, China; 3 School of Agricultural and Biological Engineering, Foshan University, Foshan 528231, China

**Keywords:** *Weissella confusa*, salt stress, intracellular metabolites, extracellular metabolites, stress response

## Abstract

Background: *Weissella confusa* is a member of the lactic acid bacterium group commonly found in many salt-fermented foods. Strains of *W. confusa* isolated from high-salinity environments have been shown to tolerate salt stress to some extent. However, the specific responses and mechanisms of *W. confusa* under salt stress are not fully understood. Methods: To study the effect of NaCl stress on *W. confusa*, growth performance and metabolite profiles of the strains were compared between a NaCl-free group and a 35% NaCl-treated group. Growth performance was assessed by measuring viable cell counts and examining the cells using scanning electron microscopy (SEM). Intracellular and extracellular metabolites were analyzed by non-targeted metabolomics based on liquid chromatography-mass spectrometry (LC-MS). Results: It was found that the viable cell count of *W. confusa* decreased with increasing salinity, and cells could survive even in saturated saline (35%) medium for 24 h. When exposed to 35% NaCl, *W. confusa* cells exhibited surface pores and protein leakage. Based on the Kyoto Encyclopedia of Genes and Genomes (KEGG) analysis, 42 different metabolites were identified in the cells and 18 different metabolites in the culture medium. These different metabolites were mainly involved in amino acid metabolism, carbohydrate metabolism, and nucleotide metabolism. In addition, salt-exposed cells exhibited higher levels of intracellular ectoine and lactose, whose precursors, such as aspartate, L-2,4-diaminobutanoate, and galactinol, were reduced in the culture medium. Conclusions: This study provides insight into the metabolic responses of *W. confusa* under salt stress, revealing its ability to maintain viability and alter metabolism in response to high NaCl concentrations. Key metabolites such as ectoine and lactose, as well as changes in amino acid and nucleotide metabolism, may contribute to its tolerance to salt. These findings may improve our understanding of the bacterium’s survival mechanisms and have potential applications in food fermentation and biotechnology.

## 1. Introduction

*Weissella confusa*, a member of the lactic acid bacteria, is frequently isolated from spontaneously fermented foods, including cereals, vegetables, dairy products, fish, and meat-based products [1]. This species exhibits a wide range of functional properties, such as the production of organic acids and exopolysaccharides, antimicrobial activity, probiotic effects, and antioxidant activity [1,2]. These strains are commonly used as starter cultures in food fermentation processes, helping to improve the safety, nutritional value, and sensory attributes of food products [1]. However, certain strains of *W. confusa* have also been identified as potential pathogens involved in the pathogenesis of various diseases [3,4]. Therefore, it is crucial to carefully evaluate the safety profiles of *W. confusa* strains before selecting them as starters for food fermentation [4,5].

In various food fermentation processes, high salinity is a critical environmental factor, especially in the production of soy sauce, fish sauce, cheese, kimchi, and sausage [1,6,7,8,9]. In particular, during the fermentation of soy sauce and fish sauce, salt concentrations can reach levels of 17–30% (*w*/*w*) [10]. Normally, high salt concentrations in these fermented products cause salt stress to microorganisms, which can affect their metabolic functions and growth, reduce fermentation efficiency, and affect the quality of the final product. *W. confusa* is commonly found in high-salt fermented foods, such as soy sauce, and is therefore exposed to significant salt stress during their production. Previous studies have shown that *W. confusa* exhibits a marked tolerance to NaCl stress, with the ability to grow at NaCl concentrations ranging from 0% to 10% (*w*/*v*) [11,12]; however, its growth is significantly inhibited at 18% (*w*/*v*) NaCl [12]. Based on the salt requirement of microorganisms, they can be divided into two types: halophilic and halotolerant [13]. *W. confusa* was identified as halotolerant because it is able to thrive in both saline and non-saline conditions. However, the specific responses and mechanisms of *W. confusa* under salt stress remain poorly understood.

In many microorganisms, two main strategies are used to cope with salt stress: the “salt-in” strategy and the “salt-out” strategy [14]. The “salt-in” strategy maintains osmotic balance by accumulating high intracellular concentrations of specific ions, primarily potassium (K^+^) [14,15]. Conversely, the “salt-out” strategy, also known as the compatible solute strategy, maintains osmotic balance by accumulating higher intracellular concentrations of compatible osmolytes [14]. Microorganisms that use the “salt-out” strategy generally exhibit greater flexibility and adaptability to salt stress than those that use the “salt-in” strategy [15]. The compatible osmolytes involved in the “salt-out” strategy, also known as osmoprotectants, are highly soluble, low molecular weight organic molecules [16]. Based on their chemical structures, the primary osmoprotectants can be categorized into amino acids, sugars, polyols, and their derivatives [16,17]. Within microbial cells, compatible solutes can be synthesized endogenously or imported from the external growth medium [17]. However, the utilization of compatible solutes in response to salt stress has been reported to be species-specific [18].

Metabolomics serves as a powerful technique to investigate the production of compatible solutes by microorganisms in response to salt stress [14]. The osmotic solute metabolism of various bacterial species has been extensively studied. For example, He et al. [19] showed that *Tetragenococcus halophilus* cells subjected to salt stress exhibited increased levels of compatible solutes such as proline, glycine, citrulline, and *N*-acetyltryptophan. Similarly, Sévin et al. [18] investigated the global metabolic responses to salt stress in twelve bacterial species, including *Bacillus subtilis*, *Corynebacterium glutamicum*, and *Lactobacillus casei*. Their study identified a range of potentially accumulating osmoprotectants, such as trehalose, glutamate, hypotaurine, malate, and *N*-acetylornithine [18]. While these studies primarily focus on intracellular modifications associated with salt stress adaptation, it is important to emphasize that microbes also release specific metabolites into the extracellular environment under such conditions [14]. These secreted metabolites, known as the exometabolome or metabolic footprint, provide valuable insights into microbial metabolic responses to environmental fluctuations [20,21]. Behrends et al. [22] investigated the exometabolome of *Pseudomonas aeruginosa* under salt stress conditions and identified changes in osmoprotectants such as glycine-betaine, trehalose, and glutamate. However, the investigation of changes in extracellular metabolites has been less thorough compared to the investigation of intracellular metabolites.

In this study, we investigated the effects of salt stress on the growth performance and metabolomic profiles of *W. confusa*. By performing a comprehensive analysis of both intracellular and extracellular metabolites, we characterized the metabolic responses to salt stress. The results of this research are expected to improve our understanding of the tolerance mechanisms in *W. confusa* and may help to identify protective agents that enhance cellular stress resistance, thereby optimizing their use in traditional fermented foods.

## 2. Materials and Methods

### 2.1. Strain and Medium Conditions

The strain used in this study, *Weissella confusa* CGMCC 21637, was isolated from a koji sample traditionally used in soy sauce fermentation in China. First, the strain was cultured statically for 10 h at 37 °C in de Man, Rogosa, and Sharpe (MRS) broth (Guangdong Huankai Microbial Sci & Tech., Co., Ltd., Guangzhou, China) to prepare seed cultures. These seed cultures were then transferred to fresh MRS medium and statically cultured for another 5 h to produce precultures.

### 2.2. Assessment of W. confusa Growth Under Salt Stress

To evaluate the growth performance of *W. confusa* under conditions of salt stress conditions, precultures were inoculated with a 10% (*v*/*v*) inoculum into fresh MRS medium containing different concentrations of NaCl (0%, 9%, 18%, 27%, and 35% *w*/*v*). After incubation for specified times, cells were serially diluted and plated onto MRS agar plates, which were then incubated at 37 °C for 24 h. The colony-forming units (CFU) were counted, and the results were expressed as log_10_ CFU/mL. All experiments were performed in triplicate.

### 2.3. Morphological Analysis of W. confusa Under Salt Stress

The morphological characteristics of *W. confusa* strains exposed to both salt and non-salt conditions were examined by scanning electron microscopy (SEM). Cells were cultured in MRS medium containing either 0% or 35% (*w*/*v*) NaCl for 24 h and then harvested by centrifugation at 8000× *g* for 3 min at 4 °C. The resulting bacterial pellet was washed three times with phosphate-buffered saline (PBS) and then fixed with 2.5% glutaraldehyde at 4 °C overnight. After fixation, the cells underwent a series of dehydration steps with 30%, 50%, 70%, 90%, and 100% (*v*/*v*) ethanol, followed by treatment with a 1:1 (*v*/*v*) mixture of ethanol and tert-butanol, and then with absolute tert-butanol, each for 15 min. The samples were then dried, sputter-coated with gold, and examined for bacterial morphology by SEM (Quattro S, Thermo Fisher Scientific, Waltham, MA, USA).

### 2.4. Determination of Protein Leakage

The leakage of the proteins was determined as it is described in the literature [23]. Cells were cultured for 24 h in PBS containing either 0% or 35% (*w*/*v*) NaCl. After centrifugation of the cell suspension (1 mL) at 8000× *g* for 10 min, the supernatant was filtered through a 0.22 μm filter membrane. The supernatant was measured for protein content using a Bradford protein assay kit (Solarbio, Beijing, China).

### 2.5. Sample Preparation for Metabolomics Analysis

For metabolomics analysis, cells were cultured in an MRS medium containing either 0% or 35% (*w*/*v*) NaCl. After 24 h of cultivation, the culture broth was collected and centrifuged at 8000× *g* for 10 min at 4 °C. The resulting cell pellet was washed three times with PBS to obtain the intracellular fraction. Simultaneously, the supernatant was filtered through a 0.22 μm membrane to obtain the extracellular fraction. Intracellular fractions were prepared according to the following protocol. Cells were first frozen in liquid nitrogen, accurately weighed to 50 mg, and transferred to a 2.0 mL centrifuge tube. A 6 mm diameter grinding bead and 400 μL of a methanol-water solution (4:1, *v*/*v*) were then added to facilitate the extraction of intracellular metabolites. The mixture was subjected to grinding for 6 min at −10 °C (50 Hz) using a frozen tissue grinder, followed by ultrasonic extraction for 30 min at 5 °C (40 kHz). The samples were then incubated at −20 °C for 30 min and then centrifuged at 13,000× *g* for 15 min at 4 °C. The supernatants were collected for the analysis of intracellular metabolites. Extracellular fractions were prepared as follows: 200 μL of extracellular fraction supernatant was mixed with 400 μL of an acetonitrile-methanol solution (1:1, *v*/*v*) in a 1.5 mL centrifuge tube. The samples were then vortexed for 30 s and subjected to ultrasonic extraction for 30 min at 5 °C (40 kHz). The samples were then incubated at −20 °C for 30 min and centrifuged at 13,000× *g* for 15 min at 4 °C. The resulting supernatant was then dried under a nitrogen gas stream. The sample was then resolubilized in 100 µL of an acetonitrile-water solution (1:1, *v*/*v*) and subjected to ultrasonic extraction for 5 min at 5 °C (40 kHz), followed by centrifugation at 13,000× *g* for 10 min at 4 °C. The supernatants were collected for analysis of extracellular metabolites.

### 2.6. Liquid Chromatography-Mass Spectrometry (LC-MS) Analysis

Samples were analyzed using a UHPLC-Q Exactive HF-X system (Thermo Fisher Scientific, Waltham, MA, USA) equipped with an ACQUITY HSS T3 column (2.1 × 100 mm, 1.8 μm; Waters Crop., Milford, MA, USA). The column was maintained at a temperature of 40 °C, with a flow rate of 0.4 mL/min and a sample injection volume of 3 μL. Mobile phase A consisted of 0.1% formic acid in a water/acetonitrile mixture (95:5, *v*/*v*), while mobile phase B consisted of 0.1% formic acid in an acetonitrile/isopropanol/water mixture (47.5:47.5:5, *v*/*v*/*v*). Mass spectrometry analyses were performed in both positive and negative ionization modes, covering a mass-to-charge ratio (*m*/*z*) range of 70 to 1050. The conditions for the electrospray ionization (ESI) source conditions were as follows: spray voltage set to ±3.5 kV for both ionization modes, a source temperature of 425 °C, a sheath gas flow rate of 50 arbitrary units (arb.), an auxiliary gas flow rate of 13 arb., and a normalized collision energy with a rolling voltage of 20–40–60 V for MS/MS. Data acquisition was performed using a data-dependent acquisition (DDA) mode.

### 2.7. Metabolomic Data Processing and Analysis

Initial processing of the LC-MS raw data was performed using Progenesis QI software (version 3.0, Waters Corp., Milford, CT, USA). This preprocessing workflow included baseline filtration, peak identification, integration, retention time correction, and alignment, resulting in the generation of a data matrix containing retention times, *m*/*z* values, and peak intensities. In parallel, the MS and MS/MS spectrometry data were matched against major databases, such as the human metabolome database (HMDB, http://www.hmdb.ca/, accessed on 3 March 2024) and the metlin database (https://metlin.scripps.edu/, accessed on 3 March 2024) to facilitate metabolite identification. The data were then uploaded to the majorbio cloud platform (https://cloud.majorbio.com, accessed on 17 July 2024) for further analysis [24]. Multivariate statistical analyses, including principal components analysis (PCA) and orthogonal partial least squares discriminant analysis (OPLS-DA), were performed using the R software package ropls (version 1.6.2). Metabolites exhibiting a Variable Importance in Projection (VIP) score greater than 1 in OPLS-DA, a *p*-value less than 0.05 in Student’s t-test, and an absolute fold-change greater than 1.5 were identified as significantly different. Intracellular and extracellular differential metabolites under salt stress were visualized using a volcano plot generated with the R software package ropls (version 1.6.2). Subsequently, pathway enrichment analysis for metabolic signaling pathways involving these differential metabolites was performed using the Kyoto Encyclopedia of Genes and Genomes (KEGG) database (http://www.genome.jp/kegg/, accessed on 22 July 2024).

## 3. Results

### 3.1. Effect of Salt Stress on the Growth of W. confusa

**Figure 1:**
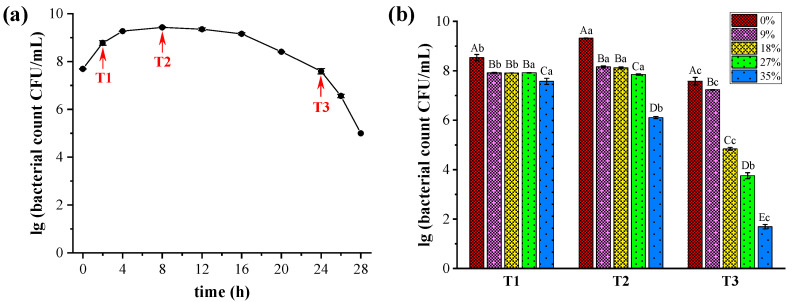
Growth performance of *Weissella confusa* under different NaCl concentrations. (**a**) Viable cell count curve of *W. confusa* treated with 0% NaCl over time. (**b**) Changes in viable cell counts of *W. confusa* exposed to different NaCl concentrations (0%, 9%, 18%, 27%, and 35%) at 2 h, 8 h, and 24 h. Error bars indicate the standard deviation of three replicate experiments. Different capital letters indicate significant differences between different NaCl concentrations at the same time point, and different lowercase letters indicate significant differences at different times in the same treatment (*p* < 0.05).

The viable cell count curve of *W. confusa* is shown in Figure 1a. In order to evaluate the tolerance of *W. confusa* to different concentrations of NaCl, investigations were carried out at three specific time points corresponding to 2 h (T1), 8 h (T2), and 24 h (T3). The cell counts of *W. confusa* cultured under different NaCl concentrations are presented in Figure 1b. At T1, the cell counts of *W. confusa* in the salt-treated group decreased to 10^7^ CFU/mL, an order of magnitude reduction compared to the control group without salt, which maintained a cell count of 10^8^ CFU/mL. In addition, no statistically significant differences (*p* > 0.05) were found among the salt-stressed groups with NaCl concentrations of 9%, 18%, and 27%. At T2, the viable bacterial counts showed different responses to different NaCl concentrations. Specifically, the groups treated with 9% and 18% NaCl showed an increase to 10^8^ CFU/mL. The group exposed to 27% NaCl maintained a cell count of 10^7^ CFU/mL, while the group exposed to 35% NaCl experienced a decrease to 10^6^ CFU/mL. However, all salt-stressed groups had lower counts than the 0% NaCl group, which had 10^9^ CFU/mL. At T3, viable cell counts decreased significantly with increasing salt concentration (*p* < 0.05). Overall, different NaCl concentrations significantly affected the growth of *W. confusa*. The viable cell counts in the 0%, 9%, and 18% NaCl groups peaked at T2, followed by T1, and were lowest at T3. Counts in the 27% NaCl group showed no significant difference between T1 and T2; however, both T1 and T2 counts were higher than those at T3. Cell counts in the 35% NaCl group consistently decreased with increasing incubation time. These results suggest that the growth performance of *W. confusa* is partially affected by salt stress at concentrations of 9%, 18%, 27%, and 35%. However, the strain appears to tolerate higher salinity levels and can survive for up to 24 h in a saturated salt solution (35%) of MRS medium. To gain a deeper understanding of the behavior of *W. confusa* under extreme salt stress, further studies will examine its response to 35% salt concentration over a 24 h period.

### 3.2. Changes in Cell Morphology Under Salt Stress

**Figure 2:**
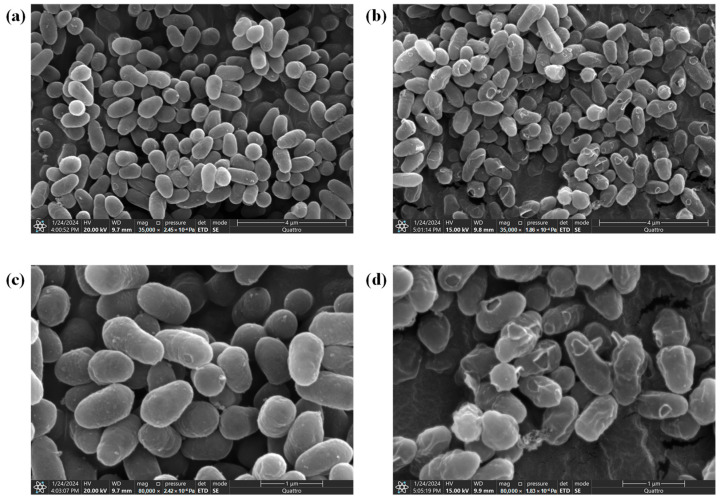
Morphological changes of *W. confusa* under NaCl conditions as viewed by scanning electron microscopy (SEM). (**a**) SEM images of *W. confusa* treated with 0% NaCl after 24 h at 35,000× magnification. (**b**) SEM images of *W. confusa* treated with 35% NaCl after 24 h at 35,000× magnification. (**c**) SEM images of *W. confusa* treated with 0% NaCl after 24 h at 80,000× magnification. (**d**) SEM images of *W. confusa* treated with 35% NaCl after 24 h at 80,000× magnification.

SEM was used to observe changes in cell morphology under 35% NaCl stress. As shown in Figure 2a,c, the majority of cells cultured in a medium without NaCl exhibited a short rod shape with a smooth surface, characteristic of *W. confusa* cells. In contrast, cells cultured in a medium containing 35% NaCl showed significant structural changes. As shown in Figure 2b,d, the cells exhibited rugose surfaces and appeared to have more surface holes. These observations suggest that high salt stress may induce morphological changes in *W. confusa*, possibly leading to the formation of surface holes.

**Figure 3:**
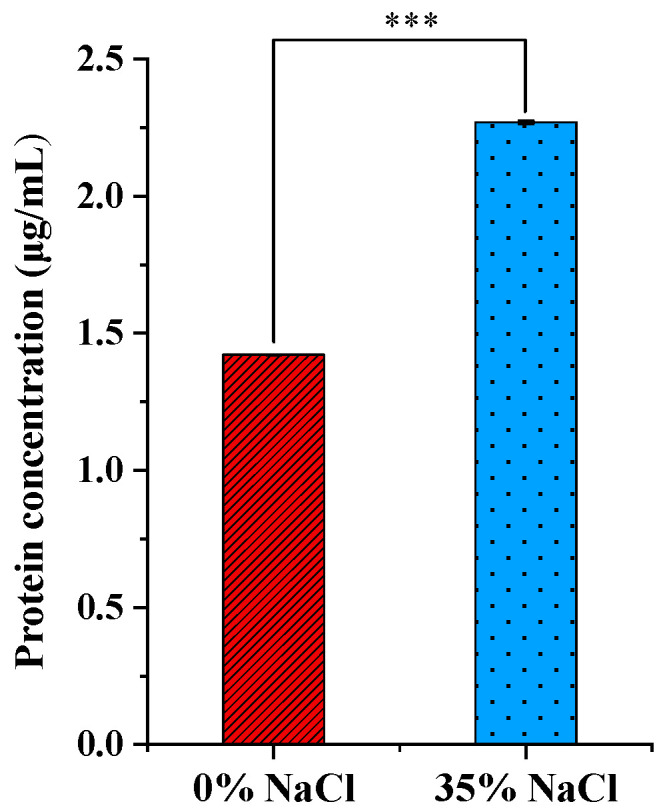
Intracellular leakage of protein in *W. confusa* after 0% and 35% NaCl treatment. Error bars indicate standard deviation of three replicate experiments. *** indicates *p*-value < 0.001.

To assess the leakage of macromolecules to the external environment under 35% NaCl conditions, the release of intracellular proteins was monitored. As shown in Figure 3, the concentration of extracellular proteins increased significantly (*p* < 0.001) in the salt-treated group compared to the salt-free group. Together with the SEM analysis, these results suggest that exposure to high salt stress may cause damage to *W. confusa* cells, possibly resulting in the formation of surface holes and protein leakage.

### 3.3. Metabolic Profiling Analysis of W. confusa Under Salt Stress

The effect of high salt stress on the metabolic profile of *W. confusa* was investigated by metabolomic analysis, which included the assessment of metabolites from both cellular and extracellular environments. After data processing and metabolite identification, a total of 1423 metabolites were detected in the cells (854 in positive ion mode and 569 in negative ion mode) (Table S1), while 2799 metabolites were identified in the culture medium (1579 in positive ion mode and 1220 in negative ion mode) (Table S2). Interestingly, a greater variety of metabolites were detected in the negative ion mode compared to the positive ion mode, both within the cells and in the culture medium. This preference suggests that *W. confusa* metabolites may be more easily ionized and detected in the negative ion mode under experimental conditions.

**Figure 4:**
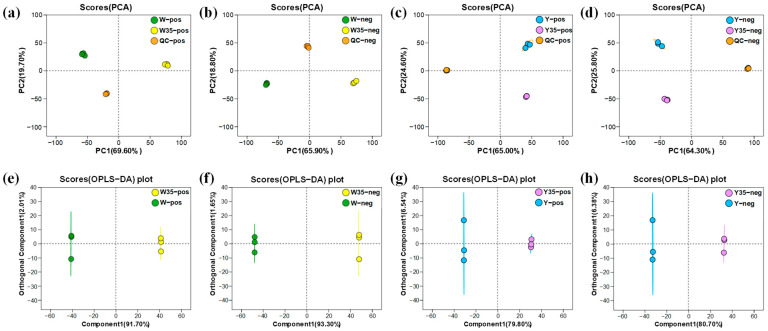
Metabolic profiling analysis of *W. confusa* between control and 35% NaCl treatment groups. (**a**) Principal components analysis (PCA) score plots of intracellular metabolic profiling in positive mode. (**b**) PCA score plots of intracellular metabolic profiling in negative mode. (**c**) PCA score plots of extracellular metabolic profiling in positive mode. (**d**) PCA score plots of extracellular metabolic profiling in negative mode. (**e**) Orthogonal partial least squares discriminant analysis (OPLS-DA) score plots of intracellular metabolic profiling in positive mode. (**f**) OPLS-DA score plots of intracellular metabolic profiling in negative mode. (**g**) OPLS-DA score plots of extracellular metabolic profiling in positive mode. (**h**) OPLS-DA score plots of extracellular metabolic profiling in negative mode. Abbreviations: W, 0% NaCl treatment groups of intracellular metabolites; W35, 35% NaCl treatment groups of intracellular metabolites; Y, 0% NaCl treatment groups of extracellular metabolites; Y35, 35% NaCl treatment groups of extracellular metabolites; QC, quality control; pos, positive ion mode; neg, negative ion mode.

PCA and OPLS-DA were used to investigate the differences between *W. confusa* samples under different conditions. The quality control (QC) samples showed a high degree of clustering in the PCA score plot, indicating good data quality and reproducibility of the analytical methods used (Figure 4a–d). The PCA model of intracellular metabolites showed a clear separation between salt-stressed and unstressed cells in both positive ion mode (Figure 4a) and negative ion mode (Figure 4b). Similarly, extracellular metabolites showed comparable separation trends (Figure 4c,d). These results indicate that salt stress induces significant changes in both intracellular and extracellular metabolites. To further investigate the metabolic differences between these groups, OPLS-DA was used. The OPLS-DA score plots for intracellular and extracellular substances showed significant separation between salt-stressed and unstressed groups in both ion modes (Figure 4e–h). The R^2^X and Q^2^ values (Table S3) indicated that the four models were stable and showed good predictive ability. The results of the PCA and OPLS-DA analyses suggested that the model was meaningful and suitable for subsequent screening of differential metabolites.

### 3.4. Screening and Identification of Differential Metabolites

**Figure 5:**
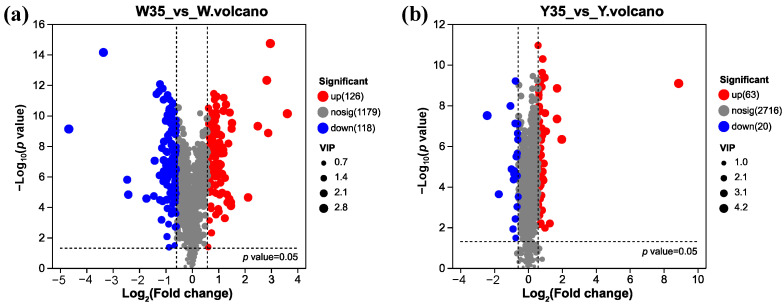
Volcano map of intracellular (**a**) and extracellular (**b**) differential metabolites in *W. confusa* between the 35% NaCl treatment group and the 0% NaCl treatment group. Each point in the volcano map represents a metabolite; the size of the points represents the VIP value of the OPLS-DA model; red points represent significantly up-regulated metabolites, blue points represent significantly down-regulated metabolites, and grey points represent non-significantly different metabolites (*p* < 0.05).

Differential metabolites were further screened by integrating the VIP score of ≥1 from the OPLS-DA model, a *p*-value of <0.05, and an absolute fold change of >1.5. In intracellular samples, a total of 244 metabolites were significantly altered in the salt-added group compared to the control group, including 99 metabolites from the positive ion model and 145 metabolites from the negative ion model (Table S4). These metabolites were visualized using a volcano plot (Figure 5a), which showed that 126 metabolites were up-regulated and 118 metabolites were down-regulated. Similarly, in the extracellular samples, 83 metabolites showed significant changes between the salt-added and control groups, with 35 metabolites detected in the positive ion mode and 48 in the negative ion mode (Table S5). This is illustrated in another volcano plot (Figure 5b), which shows 63 up-regulated and 20 down-regulated metabolites. In both intracellular and extracellular analyses, the majority of differential metabolites were classified as organic acids (mainly amino acids), lipids, and nucleic acids. This suggests that these metabolites were induced by salt stress and may serve as potential biomarkers, likely involved in biochemical pathways that facilitate the adaptation of *W. confusa* to salinity stress.

### 3.5. Metabolic Pathway Analysis of Differential Metabolites

**Figure 6:**
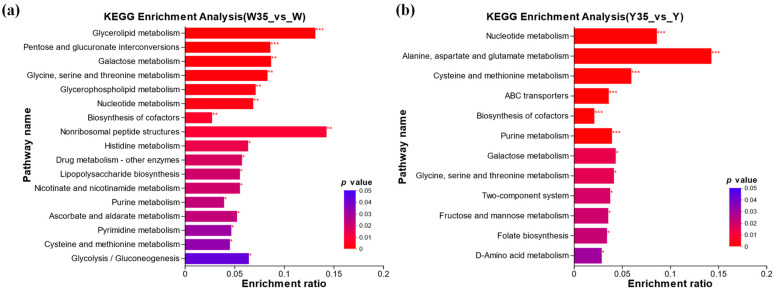
Kyoto Encyclopedia of Genes and Genomes (KEGG) enrichment analysis of intracellular (**a**) and extracellular (**b**) differential metabolites in *W. confusa* between the 35% NaCl treatment group and the 0% NaCl treatment group. The horizontal axis represents the enrichment ratio, which is the ratio of the number of differential metabolites annotated to the pathway to that of the species. The vertical axis represents the enriched KEGG pathway name. *** indicates *p*-value < 0.001, ** indicates *p*-value < 0.01, * indicates *p*-value < 0.05.

To further investigate the roles of key metabolites in *W. confusa* under salt stress, the selected significant metabolites were subjected to annotation, classification, and pathway enrichment analysis using the KEGG database. The results of the KEGG pathway enrichment analysis for differential metabolites are presented in Figure 6. The analysis revealed that intracellular differential metabolites were enriched in 76 metabolic pathways, with 17 of these pathways showing significant enrichment (*p* < 0.05, Figure 6a). These significantly enriched pathways are primarily associated with carbohydrate metabolism (including pentose and glucuronate interconversions, galactose metabolism, ascorbate and alternate metabolism, and glycolysis/gluconeogenesis), amino acid metabolism (including glycine, serine, and threonine metabolism; histidine metabolism; and cysteine and methionine metabolism), nucleotide metabolism (especially purine metabolism), and lipid metabolism (especially glycerolipid metabolism and glycerophospholipid metabolism). Extracellular differential metabolites were enriched across 26 metabolic pathways, with 12 pathways demonstrating significant enrichment (*p* < 0.05, Figure 6b). These pathways were predominantly related to amino acid metabolism (including alanine, aspartate and glutamate metabolism; cysteine and methionine metabolism; glycine, serine and threonine metabolism; and D-amino acid metabolism), carbohydrate metabolism (specifically galactose metabolism and fructose and mannose metabolism), nucleotide metabolism (specifically purine metabolism), and membrane transport (ABC transporters). Further analysis revealed five metabolic pathways, including cysteine and methionine metabolism, purine metabolism, galactose metabolism, glycine, serine, and threonine metabolism, and biosynthesis of cofactors, involving both intracellular and extracellular metabolites. Enrichment analysis results indicated that *W. confusa* employed a multifaceted strategy that included adjustments in carbohydrate, amino acid, nucleotide, and lipid metabolism to mitigate salt stress. Intracellularly, the emphasis on energy production pathways, such as glycolysis and gluconeogenesis, along with biosynthetic pathways for amino acids and nucleotides, likely underpins cellular functions under stress conditions. Extracellularly, modifications in amino acid and carbohydrate metabolism suggest potential roles in osmotic regulation and stress signaling.

### 3.6. Changes in Relative Abundances of Differential Metabolites in W. confusa Under Salt Stress

The significantly enriched KEGG pathways in the cell groups involved a total of 42 differential metabolites (Table 1), while in the culture medium groups, there were a total of 18 differential metabolites (Table 2). The pathway analysis results showed that these differential metabolites associated with salt stress were mainly related to amino acid metabolism, carbohydrate metabolism, and nucleotide metabolism. We then focused on the changes in the differential metabolites associated with these pathways.

In relation to amino acid metabolism in *W. confusa* subjected to 35% NaCl stress, notable differences were observed in eight intracellular and seven extracellular metabolites. Specifically, intracellular concentrations of ectoine and L-allothreonine were up-regulated, suggesting that salt exposure may increase their synthesis to maintain cellular osmolarity. In contrast, intracellular levels of carglumic acid, cystathionine, ergothioneine, histidinal, and 1-aminocyclopropane-1-carboxylate were down-regulated, indicating an increased utilization of these metabolites in response to salt stress. The observed down-regulation of intracellular *S*-methyl-5′-thioadenosine, coupled with its up-regulation in the extracellular medium, implies the activation of an efflux mechanism by *W. confusa* to mitigate salt stress. Alternatively, this pattern may indicate cell rupture, resulting in the release of *S*-methyl-5′-thioadenosine into the extracellular environment. Furthermore, the increased extracellular levels of citrate and *S*-adenosylhomocysteine suggest that these metabolites are overproduced in response to salt exposure. The down-regulation of extracellular aspartate, L-methionine *S*-oxide, 2-oxoglutaramate, and L-2,4-diaminobutanoate suggests that salt exposure may increase the cellular uptake of these metabolites.

In relation to carbohydrate metabolism in *W. confusa* under 35% NaCl stress, significant changes in 11 intracellular and three extracellular metabolites were observed. Specifically, the up-regulation of intracellular lactose suggests that salt exposure may result in increased lactose synthesis for the maintenance of cellular osmolality. Nine intracellular metabolites, namely D-arabitol, acetyl-CoA, threonate, phenethylamine glucuronide, UDP-glucose, UDP-galactose, D-ribulose 5-phosphate, D-glycerate 2-phosphate, and D-xylonolactone, were found to be exclusively down-regulated. This observation suggests an increased cellular consumption of these metabolites in response to salt stress. In addition, galactinol exhibited decreased levels both intracellularly and extracellularly, which may indicate a reduction in its biosynthesis or an increase in its turnover within the cells, as well as an increased uptake from the extracellular medium. Additionally, the up-regulation of extracellular L-rhamnulose suggests that it may be produced in excess in response to salt exposure, whereas the down-regulation of extracellular sorbitol suggests an enhanced cellular uptake mechanism.

In relation to nucleotide metabolism in *W. confusa* under 35% NaCl stress, eight intracellular and five extracellular metabolites exhibited significant changes. The up-regulation of intracellular dihydroorotate indicates that salt exposure may enhance its production to maintain cellular osmolarity. Seven intracellular metabolites, including cytosine deoxyribonucleoside, dGMP, adenosine diphosphate ribose, deoxyinosine, ADP-ribose, UDP, and cGMP, were exclusively down-regulated, indicating increased cellular consumption of these metabolites in response to salt stress. Correspondingly, extracellular levels of hypoxanthine, cytidine, inosine, guanosine, and ADP were up-regulated, suggesting their overproduction due to salt exposure.

In addition, nine intracellular differential metabolites of *W. confusa* exhibited significant enrichment in lipid metabolism under 35% NaCl stress. Among them, five metabolites, including CDP-DG(20:2(11Z,14Z)/18:2(9Z,12Z)), CDP-DG(i-14:0/i-15:0), LysoPC (22:6(4Z,7Z,10Z,13Z,16Z,19Z)/0:0), TG(8:0/8:0/15:0), and PA(20:1(11Z)/15:0), were up-regulated, indicating potential overproduction in response to salt exposure. In contrast, four metabolites, such as D-glycerate 2-phosphate, PA(8:0/8:0), glycerol 3-phosphate, and UDP-glucose, were down-regulated, suggesting increased cellular consumption as an adaptive response to salt stress.

## 4. Discussion

*W. confusa* is halotolerant and shows significant tolerance to NaCl stress. In the present study, the growth of *W. confusa* was assessed at different NaCl concentrations of 0%, 9%, 18%, 27%, and 35% (*w*/*v*) (Figure 1b). The results indicated a decrease in survival rates with increasing salinity levels, confirming previous research [11]. In addition, *W. confusa* demonstrated the ability to tolerate a wide range of NaCl concentrations from 0% to a saturation point of 35%. Notably, there was an initial increase in cell number at NaCl concentrations of 0%, 9%, and 18%, indicating that the strain is capable of proliferating within these specific salt concentrations. The strains used in this study were isolated from soy sauce, a traditional high salt (18%, *w*/*v*) fermented food from China, which accounts for their observed salt resistance. In particular, *W. confusa* exhibited the ability to survive in an MRS medium with 35% NaCl saturation for up to 24 h. This level of salt tolerance is atypical for strains of this type [25]. In general, bacteria that thrive in hypersaline environments, especially those approaching NaCl saturation, are extreme halophiles such as *Halorubrum tebenquichense* [13,15]. Therefore, the response of *W. confusa* to 35% NaCl stress was investigated in detail.

Morphological changes in microorganisms can serve as visible indicators of their potential adaptation to salt stress [26]. In this study, SEM images suggested that *W. confusa* cells exposed to 35% NaCl stress exhibited remarkable morphological changes, including rugose textures and the presence of holes (Figure 2). These structural alterations may indicate a disruption of the cells’ protective functions. Proteins and other cellular components are essential for cell function, and when the bacterial membrane is disrupted, these macromolecules may leak into the external environment [27]. Changes in extracellular proteins are often used to monitor membrane integrity under stress [28]. The increased extracellular proteins of *W. confusa* under salt stress could suggest damage to the cell membrane (Figure 3). The release of these biomolecules could indicate compromised membrane integrity, as the membrane normally acts as a selective barrier to retain intracellular constituents [26]. Similar morphological changes under salt stress have been observed in other microorganisms. For example, Feng et al. [29] observed that *Staphylococcus aureus* cells ruptured and released their intracellular contents after exposure to 20% NaCl.

Similarly, Hu et al. [30] reported that *Vibrio brasiliensis* cells exhibited lysis and rupture in media containing 5% and 7% NaCl. High salt concentrations are known to cause osmotic imbalance, which may lead to cellular dehydration, structural damage, the release of intracellular components, and, ultimately, cell death [31]. However, the relationship between morphological changes, protein leakage, and viable cell count of *W. confusa* under salt stress needs to be further investigated.

**Figure 7:**
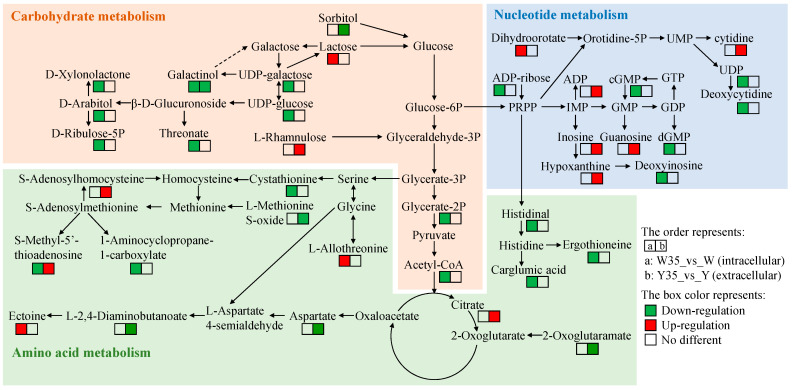
Intracellular and extracellular metabolic response of *W. confusa* to salt stress. Metabolic pathways were constructed according to the KEGG metabolic database (http://www.genome.jp/kegg/, accessed on 22 July 2024). The light orange background represents carbohydrate metabolism, the light blue background represents nucleotide metabolism, and the light green background represents amino acid metabolism. Boxes on the left side of the figure represent intracellular metabolite changes, and boxes on the right side of the figure represent extracellular metabolite changes. Boxes filled in green, red, and white represent the metabolite down-regulated, up-regulated, and no change, respectively, in treated 35% NaCl compared to 0% NaCl. Abbreviations: UDP, uridine 5′-diphosphate; UMP, uridine monophosphate; ADP, adenosine diphosphate; cGMP, cyclic guanosine monophosphate; GTP, guanosine triphosphate; PRPP, 5-phosphoribosyl-1-pyrophosphate; IMP, inosine monophosphate; GMP, guanosine monophosphate; GDP, guanosine diphosphate; dGMP, deoxyguanylic acid.

Salt stress typically induces changes in both intracellular and extracellular metabolites in microbial organisms [14]. These metabolic changes can be systematically studied using metabolomics, a technique that provides a comprehensive analysis of low molecular weight metabolites inside and outside cells [32]. To elucidate the metabolic response of *W. confusa* under salt stress, our study used metabolomics for the first time to identify changes in both intracellular and extracellular metabolites. We identified 42 significantly different metabolites in the cells and 18 in the culture medium, all of which were involved in significantly enriched KEGG pathways (Table 1 and Table 2). Previous studies have shown that salt stress induces metabolite changes, leading to the production of osmoprotectants such as proline, glycine, glycine betaine, and trehalose [18,19,22]. However, these osmoprotectants were not detected in *W. confusa* under salt stress, which may be due to factors such as the specific salt concentration [33,34], treatment duration [33,35], or species-specific differences [18]. Subsequent analysis revealed six metabolic pathways involving both intracellular and extracellular differential metabolites are jointly involved (Figure 6). In particular, we focused on amino acid metabolism, carbohydrate metabolism, and nucleotide metabolism, where the enrichment of differential metabolites is notably high. These results suggested a model for metabolic changes during salt stress (Figure 7).

Amino acid metabolism in microorganisms plays a central role in protein synthesis, energy production, stress resistance, and cell signaling [36]. Many amino acids and their derivatives act as osmoprotectants, thereby maintaining osmotic balance and facilitating the survival of microorganisms in high-salinity environments [17]. Our investigation of *W. confusa* under salt stress conditions revealed a significant enrichment of five amino acid metabolic pathways. Specifically, the pathways of glycine, serine, and threonine metabolism, as well as cysteine and methionine metabolism, exhibited significant enrichment in both intracellular and extracellular metabolites (Figure 7). Under stress conditions, glycine, serine, and threonine are critical for osmoprotectant and protein synthesis, whereas the cysteine and methionine pathways play key roles in redox regulation and methylation [15,37,38]. In this study, most of the intracellular differential metabolites enriched in the cysteine and methionine pathways were downregulated, while those in the glycine, serine, and threonine pathways were up-regulated. In addition, intracellular levels of ectoine significantly increased under salt-stress conditions. Ectoine, which is synthesized from aspartate via intermediates such as L-aspartyl phosphate, L-aspartate 4-semialdehyde, L-2,4-diaminobutanoate, and *N*(*γ*)-acetyl-diaminobutyrate, serves as a common osmoprotectant in bacteria, including *Halomonas*, *Chromohalobacter*, and *Halorhodospira* [15,39,40]. In *W. confusa*, decreased extracellular levels of aspartate and L-2,4-diaminobutanoate, both precursors for ectoine synthesis, were observed. These changes in amino acid metabolism suggest that *W. confusa* responds to NaCl-induced stress by downregulating the cysteine and methionine biosynthetic pathways, upregulating the glycine, serine, and threonine pathways, and utilizing extracellular aspartate for the production of ectoine.

Carbohydrate metabolism in microorganisms is essential for energy production and the provision of precursors for various biosynthetic pathways. Under conditions of salt stress, this metabolic process becomes critical not only for energy supply but also for ion transport, redox homeostasis, and osmotic regulation, thereby facilitating the survival of microorganisms in saline environments [25]. In our investigation of *W. confusa* under salt stress, we observed a significant enrichment of five carbohydrate metabolism pathways. The majority of intracellular metabolites associated with carbohydrate metabolism were down-regulated, suggesting a reduced requirement for reducing power and metabolic intermediates as bacterial viability decreases under salt stress conditions. In particular, galactose metabolism was enriched in both intracellular and extracellular pathways (Figure 7). Specifically, a reduction in galactinol levels was observed both intracellularly and extracellularly, whereas intracellular lactose levels increased significantly under salt stress. Galactinol, a common osmoprotectant in plants, plays a crucial role in the synthesis of raffinose family oligosaccharides, which are essential for stress management [16,41,42]. Lactose, a precursor in galactinol biosynthesis, provides essential carbon for bacterial metabolism, thereby supporting energy production and enhancing stress adaptation [43,44]. Changes in carbohydrate metabolism in *W. confusa* suggest that cells respond to NaCl-induced stress by down-regulating carbohydrate metabolism, increasing the utilization of both intracellular and extracellular galactinol, and accumulating intracellular lactose.

Nucleotide metabolism in microorganisms is crucial for DNA replication, RNA synthesis, and various physiological processes [45,46]. Under salt stress, *W. confusa* showed significant changes in nucleotide metabolism, particularly within the purine and pyrimidine pathways. The majority of intracellular nucleotide metabolites were down-regulated, indicating a reduction in cell growth under salt stress conditions. These findings are consistent with the observations of Li et al. [47], who reported downregulation of purine and pyrimidine metabolic enzymes in *Lactobacillus plantarum* under salt conditions, and Sévin et al. [18], who identified a general downregulation of nucleotide biosynthetic pathways in response to salt stress. Notably, purine metabolism was affected in both intracellular and extracellular pathways (Figure 7). Specifically, intracellular levels of dGMP, ADP-ribose, deoxyinosine, and cGMP decreased under salt stress, indicating a potential impact on DNA synthesis and repair. Among these metabolites, cGMP has been reported as a signaling molecule involved in gene expression and stress responses [48,49]. For example, Cadoret et al. [50] reported that UV-B stress in *Synechocystis* sp. PCC 6803 resulted in a rapid decrease of cGMP, a molecule crucial for signal transduction and photoacclimation processes. Interestingly, our observations revealed that the extracellular concentrations of hypoxanthine, inosine, guanosine, and ADP, all of which are associated with purine metabolism, increased under salt stress in *W. confusa*, whereas their intracellular concentrations remained stable. This phenomenon suggests either accelerated intracellular metabolism or secretion via high-affinity transporters, as proposed by Pinu et al. [21]. It is noteworthy that inosine and guanosine serve as precursors for the umami compounds 5′-inosinate and 5′-guanylate. Considering the involvement of *W. confusa* in soy sauce fermentation, an increase in extracellular levels of these metabolites may contribute to the enhancement of umami flavor in soy sauce. In conclusion, changes in nucleotide metabolism suggest that *W. confusa* adapts to salt stress by downregulating nucleotide metabolism pathways, thereby reducing intracellular purine metabolites and increasing extracellular purine nucleosides. This adaptive response probably facilitates high salt tolerance and may play a role in flavor development during soy sauce fermentation.

## 5. Conclusions

In conclusion, this study investigated the effects of salt stress on the growth performance and metabolite profiles of *W. confusa*. The results indicated that NaCl concentrations ranging from 0% to 35% appeared to have a significant effect on the growth and survival of *W. confusa*. Specifically, this organism appeared to tolerate MRS medium with up to 35% NaCl saturation for as long as 24 h. However, exposure to 35% NaCl stress resulted in the development of surface pores in the cells and protein leakage. In addition, intracellular and extracellular metabolomic analyses suggested distinct metabolic changes in response to salt stress in *W. confusa*. In response to this stress, *W. confusa* may have engaged in a broad regulatory response, with changes observed in amino acid, carbohydrate, and nucleotide metabolism. These results provide valuable insights into the cellular stress response and offer a better understanding of the mechanisms that may underlie salt tolerance.

## Figures and Tables

**Table 1:** Differential intracellular metabolites in *W. confusa* between the 35% NaCl treatment group (W35) and the 0% NaCl treatment group (W) based on significantly enriched KEGG pathways (*p* < 0.05).

Metabolite ^1^	Retention Time (min)	Ion Mode ^2^	*m*/*z* ^3^	VIP ^4^	*p*-Value ^5^	FC ^6^	W35/W ^7^
Cystathionine	0.6044	pos	223.0744	2.17	***	0.51	down
Histidinal	0.6199	pos	122.0713	1.60	***	0.61	down
L-Allothreonine	0.6277	pos	164.0292	1.84	***	1.79	up
CDP-DG(i-14:0/i-15:0)	0.6667	pos	467.7344	1.88	***	1.73	up
Ergothioneine	0.6667	pos	230.0956	1.71	***	0.64	down
Lactose	0.7440	pos	360.1496	1.87	***	1.95	up
Cytosine deoxyribonucleoside	2.7059	pos	477.1706	2.23	***	0.36	down
Phenethylamine glucuronide	2.7686	pos	298.1280	2.16	***	0.15	down
S-Methyl-5′-thioadenosine	3.0436	pos	298.0963	1.96	***	0.48	down
CDP-DG (20:2(11Z,14Z)/18:2(9Z,12Z))	3.4389	pos	515.7796	1.91	***	1.89	up
5′-Deoxy-5′-fluorouridine	3.7770	pos	229.0639	1.61	***	0.62	down
Microcystin-LR	3.9657	pos	509.2760	2.34	**	2.47	up
Notoginsenoside C	4.0675	pos	582.3001	1.91	***	1.75	up
Galactinol	0.6007	neg	341.1091	1.67	***	0.65	down
D-Glycerate 2-phosphate	0.6136	neg	166.9745	1.58	***	0.60	down
D-Arabinose 5-phosphate	0.6136	neg	229.0118	1.42	***	0.65	down
Glycerol 3-Phosphate	0.6136	neg	171.0059	1.61	***	0.62	down
UDP-galactose	0.6215	neg	565.0480	2.13	***	0.49	down
Uridine diphosphate-N-acetylglucosamine	0.6294	neg	606.0747	2.01	**	0.49	down
UDP-glucose	0.6294	neg	603.0038	1.60	***	0.63	down
Carglumic acid	0.6294	neg	189.0513	1.46	***	0.60	down
Threonate	0.6452	neg	135.0291	1.42	***	0.66	down
ADP-ribose	0.6532	neg	558.0634	2.11	***	0.43	down
D-Arabitol	0.6452	neg	197.0663	1.31	**	0.65	down
D-Ribulose 5-Phosphate	0.7248	neg	229.0118	1.48	***	0.60	down
D-Xylonolactone	0.7726	neg	295.0672	1.92	***	0.44	down
cGMP	0.8609	neg	344.0402	1.44	***	0.65	down
Dihydroorotate	0.8689	neg	157.0249	1.44	***	1.68	up
Deoxyinosine	0.8928	neg	251.0773	1.98	***	0.41	down
Adenosine diphosphate ribose	0.9936	neg	558.0644	1.94	***	0.46	down
dGMP	1.1009	neg	693.1191	2.02	***	0.39	down
NADPH	1.9828	neg	744.0840	1.97	***	0.32	down
Ectoine	1.9939	neg	343.1625	1.68	***	1.79	up
UDP	1.9883	neg	402.9949	1.59	***	0.55	down
NADH	1.9939	neg	664.1176	1.89	***	0.56	down
Acetyl-CoA	3.4394	neg	403.5557	1.73	***	0.57	down
1-Aminocyclopropane-1-carboxylate	3.6631	neg	302.1359	1.55	***	0.58	down
PA(8:0/8:0)	3.8396	neg	445.1942	1.52	***	0.59	down
PA (20:1(11Z)/15:0)	5.8044	neg	725.4572	1.54	***	1.57	up
LysoPC (22:6(4Z,7Z,10Z,13Z,16Z,19Z)/0:0)	5.8706	neg	588.3075	1.96	***	2.58	up
Nicotinic acid	6.0046	neg	368.0891	1.61	***	0.56	down
TG (8:0/8:0/15:0)	6.9767	neg	567.4630	1.60	***	1.68	up

^1^ UDP: uridine 5′-diphosphate; ADP-ribose: adenosine diphosphate ribose; cGMP: cyclic guanosine monophosphate; dGMP: deoxyguanylic acid; NADPH: nicotinamide adenine dinucleotide phosphate; NADH: nicotinamide adenine dinucleotide. ^2^ pos: positive ion mode; neg: negative ion mode. ^3^ *m*/*z*: mass-to-charge ratio. ^4^ VIP: variable importance in projection score from the orthogonal partial least squares discriminant analysis model. ^5^ ***: *p*-value < 0.001; **: *p*-value < 0.01. ^6^ FC: fold change in 35% NaCl group/0% NaCl group. ^7^ down: down-regulated, up: up-regulated.

**Table 2:** Differential extracellular metabolites in *W. confusa* between the 35% NaCl treatment group (Y35) and the 0% NaCl treatment group (Y) based on significantly enriched KEGG pathways (*p* < 0.05).

Metabolite ^1^	Retention Time (min)	Ion Mode ^2^	*m*/*z* ^3^	VIP ^4^	*p*-Value ^5^	FC ^6^	Y35/Y ^7^
L-2,4-diaminobutyric acid	0.4691	pos	163.0452	3.16	***	0.30	down
L-Rhamnulose	0.6176	pos	197.1013	2.54	***	1.54	up
Hypoxanthine	1.9202	pos	137.0460	2.69	***	1.67	up
Phenethylamine glucuronide	2.1620	pos	298.1289	3.81	***	0.19	down
S-Methyl-5′-thioadenosine	2.4192	pos	298.0970	3.01	***	1.81	up
Galactinol	0.5909	neg	387.1144	2.82	***	0.59	down
Aspartate	0.6062	neg	132.0291	2.70	***	0.64	down
Sorbitol	0.6301	neg	181.0709	2.93	***	0.62	down
L-Methionine S-oxide	0.6366	neg	164.0377	2.80	***	0.55	down
2-Oxoglutaramate	0.7200	neg	190.0349	2.75	***	0.56	down
Cytidine	0.8632	neg	278.0546	2.40	**	1.61	up
ADP	0.9786	neg	426.0221	2.63	***	1.57	up
Citrate	1.2829	neg	191.0189	3.07	***	1.56	up
7,8-Dihydropteroic acid	1.5562	neg	295.0933	2.66	***	1.66	up
S-Adenosylhomocysteine	1.9867	neg	383.1142	2.45	***	1.52	up
Guanosine	2.1072	neg	282.0842	3.03	***	1.87	up
Biopterin	2.4188	neg	282.0841	2.75	***	1.72	up
Inosine	2.4254	neg	267.0732	2.71	***	1.82	up

^1^ ADP: adenosine diphosphate. ^2^ pos: positive ion mode; neg: negative ion mode. ^3^ *m*/*z*: mass-to-charge ratio. ^4^ VIP: variable importance in projection score from the orthogonal partial least squares discriminant analysis model. ^5^ ***: *p*-value < 0.001; **: *p*-value < 0.01. ^6^ FC: fold change in 35% NaCl group/0% NaCl group. ^7^ down: down-regulated, up: up-regulated.

## Data Availability

The original contributions presented in the study are included in the article and Supplementary Material, and further inquiries can be directed to the corresponding authors.

## Supplementary Materials

The following supporting information can be downloaded at: https://www.mdpi.com/article/10.3390/metabo14120695/s1, Table S1: Intracellular metabolites in *W. confusa* under 0% and 35% NaCl conditions; Table S2. Extracellular metabolites in *W. confusa* under 0% and 35% NaCl conditions; Table S3: Quality parameters of OPLS-DA models; Table S4: Differential intracellular metabolites in *W. confusa* between the 35% NaCl treatment group (W35) and the 0% NaCl treatment group (W); Table S5: Differential extracellular metabolites in *W. confusa* between the 35% NaCl treatment group (Y35) and the 0% NaCl treatment group (Y).
