# Supplementary material for: Intracellular and Extracellular Metabolic Response of the Lactic Acid Bacterium Weissella confusa Under Salt Stress
Source: Metabolites. 2024 Dec 10;14(12):695. doi: 10.3390/metabo14120695 (PMC11678134; doi:10.3390/metabo14120695)
Supplement: Supplementary file 1 [file metabolites-14-00695-s001.zip › Table S3.pdf]

Table S3. Quality parameters of OPLS-DA models.

Samples	Ion modes	R^2X (cum) [1]	R^2Y (cum) [2]	Q^2 (cum) [3]
W35 vs W	positive	0.938	1	1
W35 vs W	negative	0.950	1	1
Y35 vs Y	positive	0.863	1	0.998
Y35 vs Y	negative	0.870	1	0.997

[1] R^2X represents the explanatory rate of the model to x.

[2] R^2Y represents the explanatory rate of the model to y.

[3] Q^2 represents the predictive ability of the model.
